# Removal of the Body of Dr. John T. Hunter

**Published:** 1860-05

**Authors:** 


☞ The body of Dr. John Hunter, who died October 16, 1793, aged 64 years, has been removed from its original resting place, and re-intered in Westminster Abbey. Steps have been taken, by the profession in England, to secure the erection of a monument over his grave, in honor of a name to which the *world owes so much.*

